# Leukocyte Counts and Ratios Are Predictive of Stroke Outcome and Hemorrhagic Complications Independently of Infections

**DOI:** 10.3389/fneur.2020.00201

**Published:** 2020-04-03

**Authors:** Aurora Semerano, Davide Strambo, Gianvito Martino, Giancarlo Comi, Massimo Filippi, Luisa Roveri, Marco Bacigaluppi

**Affiliations:** ^1^Neuroimmunology Unit, Division of Neuroscience, Institute of Experimental Neurology, San Raffaele Hospital, Milan, Italy; ^2^Neurology Department, San Raffaele Hospital, Milan, Italy; ^3^Service of Neurology, Department of Clinical Neurosciences, Lausanne University Hospital and University of Lausanne, Lausanne, Switzerland

**Keywords:** ischemic stroke, leukocytes, neutrophil to lymphocyte ratio, infection, outcome

## Abstract

**Background:** Ischemic stroke patients show alterations in peripheral leukocyte counts that may result from the sterile inflammation response as well as the occurrence of early infections. We here aimed to determine whether alterations of circulating leukocytes in acute ischemic stroke are associated with long-term functional outcome and hemorrhagic complications, independently of the occurrence of infections.

**Methods:** Blood laboratory values of patients with acute ischemic stroke, presenting within 4.5 h from symptom onset, were collected. Leukocyte subsets were analyzed in relation to 3-month functional outcome, mortality, and parenchymal hemorrhagic transformation (PH). A multivariable logistic regression analysis, considering the occurrence of early post-stroke infections, was performed for each outcome measure.

**Results:** Five-hundred-ten patients were included in the study. Independently of infections, good functional outcome was associated with a lower neutrophil to lymphocyte ratio (NL-R, OR 0.906 [95% CI 0.822–0.998]), a higher lymphocyte count (OR 1.547 [95% CI 1.051–2.277]), a higher eosinophil count (OR 1.027 [95% CI 1.007–1.048]), and a higher eosinophil to leukocyte ratio (EoLeu-R, OR 1.240 [95% CI 1.071–1.436]) at admission. Death within 3 months was associated with higher NL-R (OR 1.103 [95% CI 1.032–1.179]) as well as with lower eosinophil counts (OR 0.909 [95% CI 0.827–0.999]). Patients developing parenchymal hemorrhagic transformation had higher neutrophil counts (OR 1.420 [95% CI 1.197–1.684]) as well as a higher NL-R (OR 1.192 [95% IC 1.088–1.305]).

**Conclusion:** Leukocyte subtype profiles in the acute phase of ischemic stroke represent a predictor of outcome independently of infections. Stroke-evoked sterile inflammation is a pathophysiological relevant mechanism that deserves further investigation.

## Introduction

Immune cell activation is among the first biological responses detected after ischemic stroke (1), revealing a tight cross-talk between the ischemic brain and the peripheral immune system. Ischemic brain injury triggers not only a local inflammatory response but also induces a systemic, sterile inflammatory reaction, through different mechanisms: the release of danger-/damage-associated molecular patterns (DAMPs) from injured and dying neurons (2), the activation of the hypothalamic-pituitary-adrenal axis with increase of cortisol concentrations (3), the direct sympathetic innervation of bone marrow (4), spleen (5), lung (6), and liver (7). As a result, after a stroke, circulating immune cells vary in number, become activated, secrete cytokines and chemokines. In turn, circulating immune cells exerts an effect on brain injury that may be either beneficial or detrimental depending on the immune response profile and the leukocyte subtype involved (8, 9). Importantly, immune cells recruited within the ischemic brain may participate in the mechanisms leading to blood brain barrier disruption and hemorrhagic transformation as well as to thrombosis (9–13). Immune alterations, such as the reduction of lymphocytes, also facilitate the occurrence of infections after stroke, establishing a detrimental vicious circle (14). In turn, infections that precede, trigger (15, 16) or follow shortly stroke onset (17) may directly trigger the changes in leukocyte counts observed in ischemic stroke and might often be responsible, themselves, for a worse stroke outcome (18).

Peripheral blood counts have thus been of interest as potential predictors of stroke outcome (19): early high neutrophil counts have been associated with increased infarct volume, stroke severity, and mortality (20, 21) and neutrophil to lymphocyte ratio (NL-R) seems to predict outcome in stroke patients (22–24), as well as symptomatic intracranial hemorrhage after thrombolysis administration (22) or mechanical thrombectomy (25, 26). However, previous studies did not consider pre-existing infections and early infections after stroke as potential confounders in evaluating the relationship between leukocyte counts and outcome association. The three-way interaction between ischemic brain injury, systemic immune response, and infections raise the question if the changes in blood leukocyte subpopulations directly affect stroke outcome or if they represent just a bystander phenomenon, with the effect on outcome mainly driven by infections.

The aim of this work was to evaluate whether leukocyte counts in the acute phase of stroke are associated, independently of the occurrence of infections, with long-term functional outcome and hemorrhagic complications.

## Materials and Methods

### Study Population

This hospital-based, retrospective, observational study was conducted at the Stroke Unit of San Raffaele Scientific Institute Milan (Italy), on consecutive patients admitted from January 2008 to December 2015 with a diagnosis of ischemic stroke, presenting within 4.5 h from stroke symptom onset. In order to eliminate the possible confounder effect of disorders that could *per se* affect the leukocyte subtype counts, patients with history of pre-existing recent infections, hematological malignancies, and recent surgery were excluded. Pre-existing recent infections were defined as suggestive symptoms (i.e., cough, dyspnea, pleuritic pain, urinary tract symptoms, etc.) and history of fever within the previous 3 weeks of stroke onset and/or determination of body temperature >37.5°C at admission. As we evaluated functional outcome as one study endpoint, patients with pre-stroke functional dependence (modified Rankin Scale, mRS ≥3) were excluded. Stroke mimics were also excluded.

For each patient we recorded: 1) demographic data (age, gender); 2) medical history and vascular risk factors (hypertension, diabetes, smoking, dyslipidemia, coronary artery disease, atrial fibrillation, previous cerebrovascular events); 3) thrombolytic treatment; 4) stroke severity assessed by the NIH Stroke Scale (NIHSS) on admission and at the discharge; 5) stroke etiology according to TOAST classification (27); 6) therapy at stroke onset (use of antiplatelets, anticoagulants, statins); 7) occurrence of post-stroke early infections, defined as body temperature ≥37.5°C in two determinations or ≥37.8°C in a single determination in patients with suggestive symptoms (i.e., cough, dyspnea, pleuritic pain, urinary tract symptoms), white blood cell count ≥11,000/mL or ≤4,000/mL, pulmonary infiltrate on chest X-rays, or cultures positive for a pathogen, within 7 days from stroke symptom onset (28); 8) laboratory values (red blood cell count, hematocrit, hemoglobin, platelet count, total white blood cell count and leukocyte subtype differential counts, serum glucose concentration, C-reactive protein) from blood drawn at admission within 48 h from stroke symptom onset. Blood cell counts were assessed with an automated hemocitometer (Sysmex XN-3000 and XN-9000, Dasit). Neutrophil to lymphocyte ratio (NL-R) and eosinophil to leukocyte ratio (EoLeu-R) were calculated as the ratio on the absolute count values. Mean time of blood collection from stroke onset was 27.54 h (standard deviation 10.96 h, range 2.20–48.00 h).

### Study Outcomes

Our three study outcomes were (i) functional outcome at 3 months assessed by the modified Rankin Scale (mRS), with good outcome defined as a mRS ≤2, obtained by in-person clinical evaluation or by a telephone interview, performed at 3 months after stroke, as part of routine clinical and research follow-up of stroke patients in our Center; (ii) death within 3 months from stroke onset; (iii) occurrence of hemorrhagic transformation classified as parenchymal hemorrhage (PH, including type 1 and type 2), defined as homogenous hemorrhage with mass effect (29), assessed on standard imaging by successive head CT or brain MR scan performed after the baseline head CT or MR scan (341 patients, 66.8%, received a control CT imaging whereas 235, 46.1%, received a MRI control imaging; last follow up imaging during the hospitalization was performed after a mean of 4.2 days after stroke onset, standard deviation 3.5; 42 patients, 8.2%, did not receive control imaging because of evidence of ischemic lesion at baseline and no worsening of the neurological conditions during the hospital stay).

This study was approved by the Institutional Review Board at San Raffaele Scientific Institute (San Raffaele Ethics Committee) and has been performed in accordance with the ethical standards laid down in the 1964 Declaration of Helsinki and its later amendments; in accordance to the Board. Subjects gave written informed consent in accordance with the Declaration of Helsinki.

### Statistical Analyses

Unadjusted analyses: Univariate tests (χ^2^ test for categorical variables, Mann-Whitney U test for continuous variables) were first used to compare clinical, neuroradiological features and cell blood counts in patients with and without good outcome, death, and parenchymal hematoma.

Adjusted analyses: To investigate the relationship of each leukocyte subtype counts with functional outcome, death, and occurrence of parenchymal hematoma, we performed different multivariable logistic regression analyse for each outcome variable. Each leukocyte subtype count was entered in a separate model as independent variable together with the parameters resulted significant at the *p* ≤ 0.10 level in the previous univariate analysis. All models resulted to include early post-stroke infections. For each leukocyte subtype count we obtained the ORs and 95% confidence intervals for the three study outcomes. For the multivariate analyses, a value of *p* ≤ 0.05 was considered significant.

Predictive values: Receiver operating characteristic (ROC) curves analysis was performed to determine the area under the curve (AUC) and 95% CI of each leukocyte subtype count. An AUC value of 0.70 or higher was considered representative of an acceptable discrimination.

To test if the presence of post-stroke infections modified the relationship between NL-R and functional outcome, an interaction term between NL-R and post-stroke infections was added to the logistic regression model with functional outcome as dependent variable. The interaction was considered relevant if the *p*-value of the interaction term was ≤ 0.05.

All statistical analyses were performed using software R, version 3.0.3 [copyright (C) 2013 The R Foundation for Statistical Computing].

## Results

Out the 510 patients included in the study, 18.2% (*n* = 93) developed post-stroke infections (characteristics are shown in Table 1). We first observed, in the unadjusted analysis, that both 3-month unfavorable outcome (mRS 3–6) and mortality were associated with higher neutrophil counts, higher neutrophil to lymphocyte ratio (NL-R), and with lower lymphocyte counts, lower eosinophils counts, and a lower eosinophil to leukocyte ratio (EoLeu-R) (Supplementary Table I). Also, stroke patients who developed parenchymal hemorrhage (PH) had higher neutrophil counts and NL-R, as well as lower lymphocyte counts, eosinophils counts, and EoLeu-R (Supplementary Table I).

**Table 1:** Characteristics of the study population.

**N. of included patients**	***n* = 510**
**Demographic characteristics**	
Age—median (IQR)	74.2 (65.7–79.7)
Male sex—*n* (%)	297 (58.2%)
**Vascular risk factors**	
Hypertension—*n* (%)	352 (69.0%)
Diabetes—*n* (%)	95 (18.6%)
Smoking—*n* (%)	135 (26.5%)
Dyslipidemia—*n* (%)	109 (21.4%)
CAD—*n* (%)	95 (18.6%)
Atrial fibrillation—*n* (%)	154 (30.1%)
Previous stroke—*n* (%)	65 (12.7%)
**Stroke severity—mean (SD)**	
Baseline NIHSS—median (IQR)	6 (3–14)
Discharge NIHSS—median (IQR)	5.5 (1–9)
**Vascular territory (OXFORD)**	
TACI—*n* (%)	78 (15.3%)
PACI—*n* (%)	222 (43.5%)
POCI—*n* (%)	62 (12.2%)
LACI—*n* (%)	72 (14.1%)
No identified lesion—*n* (%)	59 (11.6%)
Multiple territories—*n* (%)	17 (3.3%)
**Stroke etiology (TOAST)**	
Large vessels—*n* (%)	91 (17.8%)
Cardioembolic—*n* (%)	150 (29.4%)
Small vessels—*n* (%)	55 (10.8%)
Undetermined—*n* (%)	196 (38.4%)
Other causes—*n* (%)	18 (3.5%)
**Complications**	
Early post-stroke infections—*n* (%)	93 (18.2%)
**Outcome measures**	
Good 3-month outcome (mRS ≤ 2)—*n* (%)	300 (58.8%)
Death within 3 months—*n* (%)	30 (5.9%)
Hemorrhagic transformation (HT)—*n* (%)	80 (15.7%)
Parenchymal hemorrhage (PH)—*n* (%)	17 (3.3%)
**Thrombolysis—*****n*** **(%)**	196 (38.4%)
**Blood tests**	
WBC (×10^6^/mL)—mean ± SD	8.39 ± 2.80
Neutrophil (×10^6^/mL)—mean ± SD	5.78 ± 2.75
Lymphocyte (×10^6^/mL)—mean ± SD	1.76 ± 0.68
NL-R—mean ± SD	4.17 ± 4.37
Monocyte (×10^6^/mL)—mean ± SD	0.70 ± 0.27
Eosinophil (×10^6^/mL)—mean ± SD	0.14 ± 0.16
Basophil (×10^6^/mL)—mean ± SD	0.004 ± 0.019
EoLeu-R—mean ± SD	0.019 ± 0.022
RBC (×10^9^/mL)—mean ± SD	4.45 ± 0.56
Hb (g/dL)—mean ± SD	13.15 ± 1.63
Hct (%)—mean ± SD	39.73 ± 4.33
MCV (fL)—mean ± SD	89.56 ± 6.34
Hb (g/dL)—mean ± SD	13.15 ± 1.63
Platelets (×10^6^/mL)—mean ± SD	207.11 ± 55.15
MPV (fL)—mean ± SD	10.99 ± 1.08
Glucose (mg/dL)—mean ± SD	100.33 ± 39.43
CRP (mg/L)—mean ± SD	13.18 ± 20.04
**Therapy at stroke onset**	
None—*n* (%)	272 (53.3%)
Antiplatelets—*n* (%)	212 (41.6%)
Anticoagulants—*n* (%)	26 (5.1%)
Statins—*n* (%)	108 (21.2%)

*CAD, Coronary artery disease; NIHSS, NIH Stroke Scale; TACI, total anterior circulation infarct; PACI, partial anterior circulation infarct; POCI, posteriori circulation infarct; LACI, lacunar infarct; WBC, white blood cells; NL-R, Neutrophil to Lymphocyte Ratio; EoLeu-R, Eosinophil to Leukocyte Ratio; RBC, red blood cells; MCV, mean corpuscular volume; MPV, mean platelet volume, CRP, C-reactive protein; IQR, interquartile range; SD, standard deviation. All percentages are referred to the total patient number*.

To verify whether leukocyte subtype counts remained associated with outcome measures independently from the occurrence of post-stroke early infections, a model of multivariable analysis adjusted for post-stroke infections (taking into account also age, NIHSS, thrombolysis, and further variables resulted significantly associated in the univariate analysis, Supplementary Table I) was performed (detailed in Supplementary Table II).

**Figure 1:**
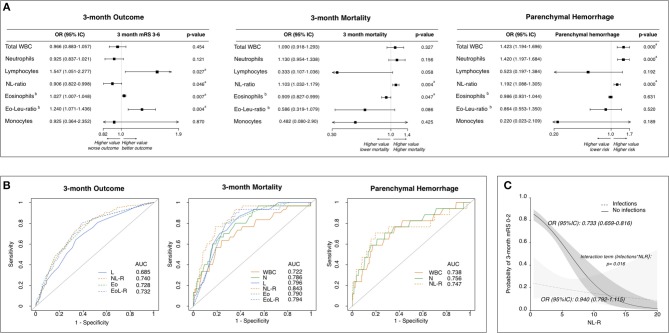
Leukocyte counts and ratios at stroke admission are predictive of stroke outcome independently of infections. **(A)** Adjusted association of leukocyte subtype counts/ratios with outcome measures. Forest plots showing leukocyte subtypes associated to 3-month functional outcome, to 3-month mortality and to parenchymal hemorrhagic transformation (Multivariate analysis). Statistics: Logistic Regression analysis. Data were adjusted for parameters resulted to be associated in the univariate analysis (Supplementary Table I) with *p* ≤ 0.10 (including post-stroke infections). Each leukocyte subtype count/ratio was entered in a separate model as independent variable (detailed in Supplementary Table II). ^a^Significant (*p* ≤ 0.05). ^b^OR is intended for 0.01-point increase of Eosinophil count and EoLeu-R. **(B)** Predictive values of leukocyte subtype counts/ratios for outcome measures. Receiver operating characteristic curves for the 3-month functional outcome, 3-month mortality and parenchymal hemorrhage. **(C)** Relationship between NL-R and stroke outcome according to post-stroke infections. Probability of good functional outcome according to NL-R in patients with (dashed line) and without (continuous line) early post-stroke infection as predicted by logistic regression model containing as independent variables: NL-R as continuous variable, early post-stroke infections as categorical dummy variable, time from stroke onset to blood sample and an interaction term between post-stroke infection and NL-R. AUC, area under curve, WBC, white blood cells; N, neutrophils; L, lymphocytes, NL-R, neutrophil to lymphocyte ratio; Eo, eosinophils; Eo-Leu-R, eosinophil to leukocyte ratio.

The multivariable analysis confirmed that, independently of infections, better functional outcome at 3 months was associated with a lower NL-R as well as with higher lymphocyte counts and higher eosinophil counts (Figure 1A). Death within 3 months was associated with higher NL-R as well as lower eosinophil counts. The development of PH was associated with higher total white blood cell (WBC) counts, neutrophil counts, and NL-R. Analyzing the predictive values of leukocyte subtypes with the outcome measures, the NL-R showed the highest predictive values for good functional outcome and mortality at 3 months (respectively 0.740 and 0.843 as area under the curve, AUC) (Figure 1B). Both neutrophil counts and NL-R had good predictive values for the development of parenchymal hemorrhage (respectively 0.756 and 0.747 AUC). Interestingly, the interaction term between NL-R and post-stroke infections resulted significant (*p* = 0.016), with a stronger relationship between NL-R and 3-month outcome in patients without infections, compared to those with post-stroke infections (OR [95%CI] = 0.733 [0.659–0.816] and 0.940 [0.792–1.115], respectively) (Figure 1C). The early determination of the NL-R is thus a valuable element in helping to predict stroke outcome in patients without stroke infections.

## Discussion

Leukocyte counts, stroke outcome and infections are pathophysiological interrelated (14, 30), with the ischemic brain injury triggering immune alterations, the immune alterations influencing post-stroke infections and outcome, and infections resulting in immune alterations and worse outcome.

Previous studies, not taking into account infections, have shown that high neutrophil counts are associated with stroke severity (20), infarct volume (21), and worse functional outcome (31). Lymphopenia has been proposed as a marker of severity of the brain damage, of a stronger stress response, and a higher risk of stroke-associated infections (32). In stroke patients, the NL-R is associated with short- and long-term outcome, and mortality (24, 33). Interestingly, in patients treated with mechanical thrombectomy, pre-treatment neutrophil counts and NL-R have been found to be independently associated to worse outcome despite reperfusion (26). In parallel, it has been recently observed that a higher NL-R is associated with Stroke Associated Pneumonia, and with pneumonia severity (34).

We here thus aimed to analyze whether leukocytes at stroke onset would predict outcome independently of the occurrence of infections. We performed a multivariable analysis adjusted for early post-stroke infections. Furthermore, patients with a history of pre-existing recent infections, hematological malignancies, recent surgery were excluded. Our analysis revealed that, even after the adjustment for the occurrence of infections, higher neutrophil counts and lower lymphocyte counts correlate with worse 3-month functional outcome, and the NL-R is associated with worse functional outcome and higher mortality. Our ROC curves analyses extend the results of previous studies (22) and show that, among the different leukocyte counts, the NL-R best predicts adverse outcome and mortality. Employing an interaction analysis, we observe a stronger relationship between NL-R and 3-month functional outcome in patients that did not develop post-stroke infections, reinforcing the concept of a direct association of a sterile immune activation with stroke outcome, independently from infections.

Being NL-R a composite parameter that resumes the information from the active inflammatory component (the neutrophils) and the adaptive regulatory arm of immunity (the lymphocytes), it represents a useful biomarker of inflammatory response. On the one hand neutrophils, early activated after sterile ischemic injury can migrate to the damaged brain tissue, accumulate in the perivascular spaces (11) and exert detrimental effects by contributing to microvessel occlusions (the “no-reflow” phenomenon) and the blood-brain-barrier damage (12). On the other hand, lymphopenia is indicative of post-stroke immunosuppression. Noteworthy also in cardiovascular diseases neutrophil counts or NL-R correlate with the severity and complexity of coronary artery disease (35), the recurrence of ischemic event (36, 37), larger myocardial infarction (38), and arterial stiffness (39).

We also tested as our third outcome measure the association of early leukocyte counts and parenchymal hemorrhage in ischemic stroke, including type 1 and type 2 PH. Indeed, we so explored possible correlations of peripheral immune alterations with haemorrhagic transformation, regardless whether it was concomitant with a clinical worsening. Other studies have shown that NL-R is associated to symptomatic intracranial hemorrhage after administration of recombinant tissue plasminogen activator (rtPA) (22) or mechanical thrombectomy (25, 26). In the present study we found that high neutrophil counts and higher NL-R values are associated to PH, independently from infections, possibly suggesting a contribution of early activated immune cells to blood brain barrier disruption or the severity of brain damage eliciting an early harsh leukocyte response (4).

In addition to NL-R, by examining comprehensive data of leukocyte subtypes, we found that high eosinophil counts are predictive of better 3-month functional outcome and lower mortality. Data exploring the prognostic role of eosinophils in the context of stroke are scarce. Reports exist of thrombotic events in patients with eosinophil-related disorders, and several hypotheses have been proposed to link eosinophilia and thrombosis. Eosinophils might indeed be a source of tissue factor, provide a pro-coagulant phospholipid surface and stimulate platelet activation by eosinophil granule contents (40). Although eosinophils might act as pro-thrombotic effectors, the association we found of lower eosinophil counts with worse outcome might instead reflect the influence of acute stroke on bone marrow granulopoiesis, favoring neutrophil over eosinophil production (41). In order to clarify the pathophysiological role of eosinophils after stroke, future studies should also regard eosinophils as a potential interesting prognostic index and further validate it.

While strengths of our study include a thorough analysis of all leukocyte subsets after stroke and strict inclusion criteria to best evaluate the relationship between leukocytes and stroke outcome by reducing confounding factors, limitations include a single time-point of blood analysis and the retrospective nature of the study. A technical limitation could also exist in the complete reliability of routinely automated counts regarding leukocyte populations with a very low number of cells, so that further analyses on eosinophil counts are warranted. Moreover, in this study, we cannot elucidate causes and effects, and further studies are needed to understand whether our observations reflect the essential systemic immune reaction triggered by the sterile ischemic brain injury (4) or a peculiar immune activation possibly linked to other factors involved in stroke onset and precipitating outcome (42, 43). Also, exploring the role of neutrophil and lymphocyte heterogeneity in the acute phase of stroke deserves further investigation.

In conclusion, neutrophil to lymphocyte ratio and eosinophil counts are capable, independently of infections, to predict outcome reinforcing the concept that profound hematopoietic alterations are induced early in stroke. Nonetheless, further analyses of leukocyte subpopulations over time after stroke are needed both to validate potential biomarkers and to understand better the stroke leuko-pathophysiology.

## Data Availability Statement

The datasets generated for this study are available on request to the corresponding author.

## Ethics Statement

The studies involving human participants were reviewed and approved by San Raffaele Ethic Committee. The patients/participants provided their written informed consent to participate in this study.

## Author Contributions

AS: data collection, data analysis, and drafting of the manuscript. DS: data analysis and drafting of the manuscript. GM, GC, MF, and LR: revision of the manuscript for content. MB: concept, drafting, and revision of the manuscript.

### Conflict of Interest

The authors declare that the research was conducted in the absence of any commercial or financial relationships that could be construed as a potential conflict of interest.
