# Supplementary material for: Leukocyte Counts and Ratios Are Predictive of Stroke Outcome and Hemorrhagic Complications Independently of Infections
Source: Front Neurol. 2020 Apr 3;11:201. doi: 10.3389/fneur.2020.00201 (PMC7145963; doi:10.3389/fneur.2020.00201)
Supplement: Supplementary file 1 [file Table_1.DOCX]

Supplementary Material

**Supplementary Table I. Association of baseline characteristics and leukocyte counts/ratios with study outcomes (Univariate analysis).**

|  | **3-month Functional Outcome** | | | **3-month Mortality** | | | **Parenchymal hemorrhage** | | |
| --- | --- | --- | --- | --- | --- | --- | --- | --- | --- |
|  | **mRS ≤2 (***n=300)* | **mRS≥3 (***n=210)* | **p** | **Alive**  *(n=480)* | **Dead**  *(n=30)* | **p** | **No**  *(n=493)* | **Yes**  *(n=17)* | **p** |
| **Demographic characteristics** |  |  |  |  |  |  |  |  |  |
| Age – mean (SD) | 71.9  (14.1) | 76.9 (12.4) | **0.000** | 73.8 (14.1) | 78 (8.4) | **0.007** | 73.9 (14) | 79.4 (4.7) | **0.080** |
| Male sex – n (%) | 183  (61%) | 114 (54.3%) | 0.155 | 284 (59.2%) | 13 (43.3%) | 0.130 | 289 (58.6%) | 8 (47.1%) | 0.484 |
| **Vascular Risk Factors** |  |  |  |  |  |  |  |  |  |
| Hypertension – n (%) | 209 (69.7%) | 143 (68.1%) | 0.779 | 331 (69%) | 21 (70%) | 1.000 | 340 (69%) | 12 (70.6%) | 1.000 |
| Diabetes – n (%) | 54 (18%) | 41 (19.5%) | 0.749 | 88 (18.3%) | 7 (23.3%) | 0.659 | 90 (18.3%) | 5 (29.4%) | 0.398 |
| Smoking – n (%) | 83 (27.7%) | 52 (24.8%) | 0.529 | 130 (27.1%) | 5 (16.7%) | 0.298 | 133 (27%) | 2 (11.8%) | 0.263 |
| Dyslipidemia – n (%) | 66 (22%) | 43 (20.5%) | 0.762 | 103 (21.5%) | 6 (20%) | 1.000 | 104 (21.1%) | 5 (29.4%) | 0.602 |
| CAD – n (%) | 50 (16.7%) | 45 (21.4%) | 0.214 | 85 (17.7%) | 10 (33.3%) | **0.059** | 91 (18.5%) | 4 (23.5%) | 0.833 |
| Atrial fibrillation – n (%) | 75 (25%) | 79 (37.6%) | **0.003** | 134 (27.9%) | 20 (66.7%) | **0.000** | 148 (30%) | 6 (35.3%) | 0.844 |
| **Stroke severity** |  |  |  |  |  |  |  |  |  |
| Baseline NIHSS– mean (SD) | 4 (5) | 14 (13) | **0.000** | 6 (9) | 17.5 (7.2) | **0.000** | 6 (10) | 17 (11) | **0.001** |
| Discharge NIHSS – mean (SD) | 2 (2) | 11 (13) | **0.000** | 3 (6) | 21.5 (28.8) | **0.000** | 3 (7) | 14 (14) | **0.000** |
| **Stroke Etiology (TOAST)** |  |  |  |  |  |  |  |  |  |
| Large vessels – n (%) | 46 (15.3%) | 45 (21.4%) | **0.000** | 87 (18.1%) | 4 (13.3%) | **0.000** | 89 (18.1%) | 2 (11.8%) | 0.399 |
| Cardioembolic – n (%) | 75 (25%) | 75 (35.7%) |  | 130 (27.1%) | 20 (66.7%) |  | 143 (29%) | 7 (41.2%) |  |
| Small vessels – n (%) | 39 (13%) | 16 (7.6%) |  | 55 (11.5%) | 0 (0%) |  | 55 (11.2%) | 0 (0%) |  |
| Undetermined – n (%) | 133 (44.3%) | 63 (30%) |  | 192 (40%) | 4 (13.3%) |  | 188 (38.1%) | 8 (47.1%) |  |
| Other causes – n (%) | 7 (2.3%) | 11 (5.2%) |  | 16 (3.3%) | 2 (6.7%) |  | 18 (3.6%) | 0 (0%) |  |
| **Complications** |  |  |  |  |  |  |  |  |  |
| Early Post-Stroke Infections – n (%) | 16 (5.3%) | 77 (36.7%) | **0.000** | 74 (15.4%) | 19 (63.3%) | **0.000** | 85 (17.2%) | 8 (47.1%) | **0.005** |
| **Haemorrhagic transformation** – n (%) | 21 (7%) | 59 (28.1%) | **0.000** | 69 (14.4%) | 11 (36.7%) | **0.003** | 63 (12.8%) | 17 (100%) | **0.000** |
| Parenchymal Hemorrhage (PH) – n (%) | 2 (0.7%) | 15 (7.1%) | **0.000** | 12 (2.5%) | 5 (16.7%) | **0.000** | 0 (0%) | 17 (100%) | **0.000** |
| **Thrombolysis** – n (%) | 105 (35%) | 91 (43.3%) | **0.070** | 185 (38.5%) | 11 (36.7%) | 0.991 | 6 (10%) | 17 (11%) | **0.001** |
| **Blood routine tests** | 206 (69.2) | 198 (66) | 0.064 | 201.5 (67.2) | 202.5 (76) | 0.787 | 202 (69) | 184 (48) | **0.057** |
| Total RBC (x 10^6^/mL) – mean (SD) | 4.5 (0.6) | 4.3 (0.6) | **0.008** | 4.4 (0.7) | 4.3 (0.8) | 0.334 | 4.4 (0.7) | 4.3 (0.5) | 0.471 |
| Hb (g/dL) – mean (SD) | 13.27 (1.6) | 12.98 (1.7) | **0.046** | 13.16 (1.6) | 13.03 (2.1) | 0.909 | 13.15 (1.6) | 13.14 (1.3) | 0.947 |
| Hct (%) - mean (SD) | 40.5 (5.5) | 39.1 (5.5) | 0.013 | 40 (5.5) | 39 (7.1) | 0.778 | 40 (5.6) | 39.9 (4.1) | 0.690 |
| MCV (fL) - mean (SD) | 89.28 (6.4) | 89.97 (6.3) | 0.149 | 89.48 (6.4) | 90.82 (4.6) | 0.283 | 89.55 (6.3) | 89.74 (8.0) | 0.576 |
| Platelets (x 10^6^/mL) – mean (SD) | 210.53 (56.3) | 202.23 (53.2) | **0.064** | 207.27 (55.4) | 204.53 (52.2) | 0.787 | 207.81 (44.1) | 187 (53.0) | **0.057** |
| MPV (fL) – mean (SD) | 11.00 (1.0) | 11.05 (1.1) | 0.454 | 11.00 (1.1) | 10.97 (0.9) | 0.861 | 10.98 (1.1) | 11.48 (1.1) | **0.058** |
| CRP (mg/L) – mean (SD) | 4.6 (8) | 11.2 (16.7) | **0.000** | 6.7 (12.1) | 20.3 (34.1) | **0.001** | 7.2 (13) | 6.4 (41.8) | 0.368 |
| Glucose (mg/dL) – mean (SD) | 86 (19) | 96 (35) | **0.000** | 89 (26) | 122 (66) | **0.001** | 89 (27) | 119.5 (84.8) | **0.002** |
| **Therapy at stroke onset** |  |  |  |  |  |  |  |  |  |
| None – n (%) | 161 (53.7%) | 111 (52.9%) | 0.398 | 263 (54.8%) | 9 (30%) | **0.030** | 265 (53.8%) | 7 (41.2%) | 0.267 |
| Antiplatelets – n (%) | 127 (42.3%) | 85 (40.5%) |  | 193 (40.2%) | 19 (63.3%) |  | 202 (41%) | 10 (58.8%) |  |
| Anticoagulants – n (%) | 12 (4%) | 14 (6.7%) |  | 24 (5%) | 2 (6.7%) |  | 26 (5.3%) | 0 (0%) |  |
| Statins – n (%) | 62 (20.7%) | 46 (21.9%) | 0.821 | 101 (21%) | 7 (23.3%) | 0.946 | 104 (21.1%) | 4 (23.5%) | 1.000 |
| **Leukocyte counts and ratios** |  |  |  |  |  |  |  |  |  |
| WBC – mean (SD) | 7.82 ±2.32 | 9.21 ±3.22 | **0.000** | 8.22 ±2.57 | 11.11 ±4.56 | **0.000** | 8.28 ±2.65 | 11.58 ±4.78 | **0.000** |
| Neutrophil – mean (SD) | 5.02 ±2.11 | 6.85 ±3.17 | **0.000** | 5.57 ±2.46 | 9.08 ±4.55 | **0.000** | 5.65 ±2.53 | 9.51 ±5.29 | **0.000** |
| Lymphocyte – mean (SD) | 1.93 ±0.68 | 1.51 ±0.60 | **0.000** | 1.79 ±0.68 | 1.15 ±0.40 | **0.000** | 1.77 ±0.67 | 1.34 ±0.81 | **0.011** |
| NL-R – mean (SD) | 3.01 ±2.07 | 5.84 ±5.97 | **0.000** | 3.79 ±3.22 | 10.26 ±11.10 | **0.000** | 3.86 ±2.96 | 13.31 ±15.69 | **0.001** |
| Monocyte – mean (SD) | 0.67 ±0.22 | 0.74 ±0.33 | **0.007** | 0.69 ±0.26 | 0.84 ±0.42 | **0.031** | 0.70 ±0.27 | 0.67 ±0.23 | 0.628 |
| Eosinophil – mean (SD) | 0.18 ±0.18 | 0.08 ±0.12 | **0.000** | 0.15 ±0.17 | 0.02 ±0.05 | **0.000** | 0.14 ±0.16 | 0.06 ±0.14 | **0.001** |
| EoLeu-R – mean (SD) | 0.025 ±0.023 | 0.011±0.016 | **0.000** | 0.020 ±0.022 | 0.003±0.008 | **0.000** | 0.020 ±0.022 | 0.007 ±0.016 | **0.002** |

*Abbreviations:* NIHSS, NIH Stroke Scale; RBC, red blood cells; MCV, mean corpuscular volume; MPV, mean platelet volume, CRP, C-reactive protein; WBC, white blood cells; NL-R, Neutrophil to Lymphocyte Ratio; EoLeu-R, Eosinophil to Leukocyte Ratio; SD, standard deviation. *Statistics***:** χ2 test for categorical variables, Mann-Whitney U test for continuous variables. Values are expressed ad mean ± SD or number (n) and percentage (%), as indicated. Values for WBC, neutrophil, lymphocyte, monocyte and eosinophil are displayed as count x 10^6^/mL.

**Supplementary Table II. Adjusted association of leukocyte subtype counts/ratio with study outcomes (Multivariate analysis).**

| **Model 1 for total White Blood Cells** | | | |
| --- | --- | --- | --- |
|  | **VARIABLES SELECTED**  **BY THE MODEL** | **OR (95%CI)** | **VARIABLES EXCLUDED BY THE MODEL** |
| **Good outcome at 3 months** | Age Thrombolysis Pre-stroke mRS Baseline NIHSS  Blood sample timing Total WBC Post-stroke early infections | 0.98 (0.96-1) 1.54 (0.92-2.59) 0.46 (0.31-0.68) 0.82 (0.78-0.86) 0.98 (0.96-1) 0.97 (0.88-1.06) 0.35 (0.17-0.71) | Atrial fibrillation, Stroke etiology (TOAST classification), Total RBC, Platelets, Hct, CRP, Glucose |
| **Death within 3 months** | Age Thrombolysis Baseline NIHSS  Blood sample timing Total WBC Glucose Post-stroke early infections Stroke etiology (LAA) Stroke etiology (SVD) Stroke etiology (OC or UND) | 1.14 (1.05-1.23) 0.81 (0.28-2.37) 1.15 (1.06-1.25) 1.02 (0.98-1.07) 1.09 (0.92-1.29) 1.01 (1-1.02) 1.77 (0.56-5.58) 0.38 (0.1-1.49) 0 (0-Inf) 0.13 (0.03-0.52) | Coronary artery disease, Atrial fibrillation, CRP |
| **Parenchymal Hemorrhage** | Age Thrombolysis Pre-stroke mRS Baseline NIHSS  Blood sample timing Total WBC Platelets Post-stroke early infections | 1.04 (0.98-1.11) 1.49 (0.49-4.5) 1.94 (1.01-3.74) 1.04 (0.96-1.13) 0.97 (0.92-1.02) 1.42 (1.19-1.7) 0.98 (0.97-1) 0.83 (0.22-3.07) | Glucose |
| **Model 2 for Neutrophils** | | | |
| **Good outcome at 3 months** | Age Thrombolysis Pre-stroke mRS Baseline NIHSS Blood sample timing Neutrophils Post-stroke early infections | 0.98 (0.96-1) 1.56 (0.93-2.62) 0.46 (0.31-0.68) 0.82 (0.79-0.86) 0.98 (0.96-1) 0.92 (0.84-1.02) 0.37 (0.18-0.76) | Atrial fibrillation, Stroke etiology (TOAST classification), Total RBC, Platelets, Hct, CRP, Glucose |
| **Death within 3 months** | Age Thrombolysis Baseline NIHSS Blood sample timing Glucose Neutrophils Post-stroke early infections Stroke etiology (LAA) Stroke etiology (SVD) Stroke etiology (OC or UND) | 1.14 (1.05-1.23) 0.77 (0.26-2.25) 1.15 (1.06-1.25) 1.02 (0.98-1.07) 1.01 (1-1.02) 1.13 (0.95-1.34) 1.65 (0.52-5.18) 0.4 (0.1-1.57) 0 (0-Inf) 0.14 (0.03-0.58) | Coronary artery disease, Atrial fibrillation, CRP |
| **Parenchymal Hemorrhage** | Age Thrombolysis Baseline NIHSS Blood sample timing Platelets Neutrophils Post-stroke early infections | 1.04 (0.98-1.11) 1.38 (0.46-4.12) 1.03 (0.95-1.11) 0.97 (0.91-1.02) 0.98 (0.97-1) 1.42 (1.2-1.68) 0.91 (0.25-3.28) | Pre-stroke mRS, Glucose |
| **Model 3 for Lymphocytes** | | | |
| **Good outcome at 3 months** | Age Thrombolysis Pre-stroke mRS Baseline NIHSS Blood sample timing Lymphocytes Post-stroke early infections | 0.98 (0.96-1) 1.48 (0.88-2.48) 0.49 (0.33-0.71) 0.82 (0.79-0.86) 0.98 (0.96-1) 1.55 (1.05-2.28) 0.36 (0.18-0.72) | Atrial fibrillation, Stroke etiology (TOAST classification), Total RBC, Platelets, Hct, CRP, Glucose |
| **Death within 3 months** | Age Thrombolysis Baseline NIHSS  Blood sample timing Glucose Lymphocytes Post-stroke early infections Stroke etiology (LAA) Stroke etiology (SVD) Stroke etiology (OC or UND) | 1.12 (1.03-1.21) 0.83 (0.29-2.39) 1.14 (1.05-1.24) 1.04 (0.99-1.09) 1.01 (1-1.02) 0.33 (0.11-1.04) 1.96 (0.64-5.97) 0.42 (0.1-1.67) 0 (0-Inf) 0.15 (0.04-0.64) | Coronary artery disease, Atrial fibrillation, CRP |
| **Parenchymal Hemorrhage** | Age Thrombolysis Baseline NIHSS Blood sample timing Lymphocytes Post-stroke early infections | 1.03 (0.97-1.09) 1.6 (0.57-4.47) 1.07 (0.99-1.15) 0.98 (0.93-1.03) 0.52 (0.2-1.38) 1.68 (0.54-5.26) | Pre-stroke mRS, Platelets, Glucose |
| **Model 4 for Neutrophil to Lymphocyte Ratio (NL-R)** | | | |
| **Good outcome at 3 months** | Age Thrombolysis Pre-stroke mRS Baseline NIHSS Blood sample timing NL-R Post-stroke early infections | 0.98 (0.96-1) 1.53 (0.92-2.57) 0.46 (0.32-0.68) 0.83 (0.79-0.87) 0.98 (0.96-1) 0.91 (0.82-1) 0.39 (0.19-0.8) | Atrial fibrillation, Stroke etiology (TOAST classification), Total RBC, Platelets, Hct, CRP, Glucose |
| **Death within 3 months** | Age Thrombolysis Baseline NIHSS Blood sample timing NL-R Post-stroke early infections | 1.05 (1-1.1) 0.6 (0.24-1.5) 1.15 (1.07-1.22) 1.02 (0.98-1.06) 1.1 (1.03-1.18) 1.75 (0.65-4.69) | Coronary artery disease, Atrial fibrillation, Stroke etiology (TOAST classification), CRP, Glucose |
| **Parenchymal Hemorrhage** | Age Thrombolysis Pre-stroke mRS Baseline NIHSS Blood sample timing Platelets NL-R Post-stroke early infections | 1.03 (0.97-1.09) 1.47 (0.45-4.78) 1.9 (0.98-3.68) 1.05 (0.97-1.14) 0.97 (0.92-1.02) 0.99 (0.98-1) 1.19 (1.09-1.3) 0.6 (0.15-2.46) | Glucose |
| **Model 5 – for Monocytes** | | | |
| **Good outcome at 3 months** | Age Thrombolysis Pre-stroke mRS Baseline NIHSS Blood sample timing Monocytes Post-stroke early infections | 0.98 (0.96-1) 1.52 (0.91-2.55) 0.46 (0.32-0.68) 0.82 (0.78-0.85) 0.98 (0.96-1) 0.93 (0.36-2.35) 0.33 (0.16-0.68) | Atrial fibrillation, Stroke etiology (TOAST classification), Total RBC, Platelets, Hct, CRP, Glucose |
| **Death within 3 months** | Age Thrombolysis Baseline NIHSS Blood sample timing Glucose Monocytes Post-stroke early infections Stroke etiology (LAA) Stroke etiology (SVD) Stroke etiology (OC or UND) | 1.14 (1.05-1.23) 0.95 (0.34-2.69) 1.16 (1.06-1.26) 1.03 (0.98-1.08) 1.01 (1-1.03) 0.48 (0.08-2.9) 2.25 (0.74-6.84) 0.37 (0.1-1.45) 0 (0-Inf) 0.11 (0.03-0.47) | Coronary artery disease, Atrial fibrillation, CRP |
| **Parenchymal Hemorrhage** | Age Thrombolysis Baseline NIHSS  Blood sample timing Glucose Monocytes Infections | 1.04 (0.98-1.11) 1.55 (0.51-4.72) 1.08 (0.99-1.17) 0.99 (0.94-1.04) 1.01 (1-1.02) 0.22 (0.02-2.11) 2.21 (0.65-7.46) | Pre-stroke mRS, Platelets |
| **Model 6 – for Eosinophils** | | | |
| **Good outcome at 3 months** | Age Thrombolysis Pre-stroke mRS Baseline NIHSS Blood sample timing Eosinophils Post-stroke early infections | 0.98 (0.96-1) 1.52 (0.91-2.56) 0.46 (0.31-0.67) 0.83 (0.79-0.87) 0.98 (0.95-1) 1.03 (1.01-1.05) 0.36 (0.17-0.73) | Atrial fibrillation, Stroke etiology (TOAST classification), Total RBC, Platelets, Hct, CRP, Glucose |
| **Death within 3 months** | Age Thrombolysis Baseline NIHSS Blood sample timing  Glucose Eosinophils Post-stroke early infections Stroke etiology (LAA) Stroke etiology (SVD) Stroke etiology (OC or UND) | 1.13 (1.04-1.22) 0.86 (0.3-2.45) 1.12 (1.03-1.21) 1.03 (0.98-1.08) 1.01 (1-1.02) 0.91 (0.83-1) 1.9 (0.64-5.68) 0.35 (0.09-1.4) 0 (0-Inf) 0.12 (0.03-0.5) | Coronary artery disease, Atrial fibrillation, CRP |
| **Parenchymal Hemorrhage** | Age IV thrombolysis Baseline NIHSS First blood sample timing  Glucose Eosinophils Post-stroke early infections | 1.04 (0.98-1.11) 1.47 (0.48-4.45) 1.06 (0.97-1.16) 0.98 (0.93-1.03) 1.01 (1-1.02) 0.99 (0.93-1.04) 1.97 (0.58-6.73) | Pre-stroke mRS, Platelets |
| **Model 7 – for Eosinophil to Leukocyte Ratio (EoLeu-R)** | | | |
| **Good outcome at 3 months** | Age IV thrombolysis Pre-stroke mRS Baseline NIHSS First blood sample timing  EoLeuR Post-stroke early infections | 0.98 (0.96-1) 1.56 (0.93-2.63) 0.45 (0.31-0.67) 0.83 (0.8-0.87) 0.98 (0.95-1) 1.24 (1.07-1.44) 0.36 (0.18-0.74) | Atrial fibrillation, Stroke etiology (TOAST classification), Total RBC, Platelets, Hct, CRP, Glucose |
| **Death within 3 months** | Age IV thrombolysis Baseline NIHSS First blood sample timing  Glucose EoLeu-R Post-stroke early infections Stroke etiology (LAA) Stroke etiology (SVD) Stroke etiology (OC or UND) | 1.13 (1.04-1.22) 0.87 (0.31-2.46) 1.12 (1.03-1.22) 1.02 (0.98-1.07) 1.01 (1-1.02) 0.59 (0.32-1.08) 1.96 (0.66-5.84) 0.35 (0.09-1.39) 0 (0-Inf) 0.12 (0.03-0.5) | Coronary artery disease, Atrial fibrillation, CRP |
| **Parenchymal Hemorrhage** | Age IV thrombolysis Baseline NIHSS First blood sample timing  Glucose EoLeu-R Post-stroke early infections | 1.04 (0.98-1.11) 1.43 (0.47-4.36) 1.05 (0.96-1.15) 0.98 (0.93-1.03) 1.01 (1-1.02) 0.86 (0.55-1.35) 1.95 (0.57-6.63) | Pre-stroke mRS, Platelets |

*Abbreviations*: NIHSS, NIH Stroke Scale; WBC, white blood cells; NL-R, Neutrophil to Lymphocyte Ratio; EoLeu-R, Eosinophil to Leukocyte Ratio; RBC, red blood cells; Hct, hematocrit; CRP, C-reactive protein; LAA, Large Artery Atherosclerosis (Large vessels); SVD, small vessels disease (Small vessels); OC, Other causes; UND, undetermined. All percentages are referred to the total patient number.
